# Tribological Performance of Anti-Wear Coatings on Tools for Forming Aluminium Alloy Sheets Used for Producing Pull-Off Caps

**DOI:** 10.3390/ma16196465

**Published:** 2023-09-28

**Authors:** Kamil Czapla, Krzysztof Żaba, Marcin Kot, Ilona Nejman, Marcin Madej, Tomasz Trzepieciński

**Affiliations:** 1 Canpack Metal Closures, ul. Kochanowskiego 28b, 33-100 Tarnów, Poland; kamil.czapla@canpack.com; 2 Department of Metal Working and Physical Metallurgy of Non-Ferrous Metals, Faculty of Non-Ferrous Metals, AGH—University of Science and Technology, al. Adama Mickiewicza 30, 30-059 Cracow, Poland; 3 Faculty of Mechanical Engineering and Robotics, AGH—University of Science and Technology, al. Adama Mickiewicza 30, 30-059 Cracow, Poland; kotmarc@agh.edu.pl; 4 Department of Materials Science and Engineering of Non-Ferrous Metals, Faculty of Non-Ferrous Metals, AGH—University of Science and Technology, al. Adama Mickiewicza 30, 30-059 Cracow, Poland; inejman@agh.edu.pl; 5 Faculty of Metals Engineering and Industrial Computer Science, AGH—University of Science and Technology, al. Adama Mickiewicza 30, 30-059 Cracow, Poland; mmadej@agh.edu.pl; 6 Department of Manufacturing Processes and Production Engineering, Faculty of Mechanical Engineering and Aeronautics, Rzeszow University of Technology, al. Powst. Warszawy 8, 35-959 Rzeszów, Poland; tomtrz@prz.edu.pl

**Keywords:** aluminium alloy, coefficient of friction, surface topography, wear, wear resistance

## Abstract

Ensuring adequate reliability of the production process of packaging closures has made it necessary to study the effect of annealing and varnishing variants on the strength and structural properties of the stock material. As a test material, EN AW-5052-H28 aluminium alloy sheets with a thickness of 0.21 mm were used. The surface treatment of the test material involved varnishing the sheet metal surface using various varnishes and soaking the sheet metal. The coefficient of friction and the abrasion resistance of the coatings were determined using the T-21 ball-and-disc tribotester. The tested sheets were subjected to tribological analysis by the T-05 roller-block tribotester using countersamples made of Caldie and Sverker 21 tool steels. The results of the tests showed differences in mechanical and structural properties depending on the method of sample preparation. Based on the test results, significant differences in the adhesion of anti-wear coatings were found. The results revealed that the most favourable friction conditions are provided by the CrN coating. The (AlTi)N interlayer in the (AlTi)N/(AlCr)N coating adheres to the substrate over the entire tested area and no detachment from its surface was observed, which proves good bonding at the substrate/coating interface. The tested AlTiN/TiAlSiXN coating is characterised by a more homogeneous, compact microstructure compared to the (AlTi)N/(AlCr)N coating.

## 1. Introduction

The possibility of shaping the properties of materials by heat treatment and the selection of chemical composition is limited for structural steel materials. Improvement to these properties is possible by applying modifications to the surface layer [1]. One possibility is to create protective coatings on the surface of the material. Physical Vapour Deposition (PVD) and Chemical Vapour Deposition (CVD) methods differ primarily in process parameters, temperature and time, as well as their mechanisms of protective layer formation [2,3]. PVD methods are practically used to cover the surfaces of tools with titanium nitride TiN (less often with titanium carbide TiC) in order to achieve an increase in their durability. The increase in the tool’s durability can be obtained as a result of complex treatments, e.g., nitriding with additional TiN coating or alternating coating with different layers of TiN + TiC [4]. The layers obtained by the PVD method have a thickness of about 3–5 μm and a hardness of about 2000–3500 HV [4]. Tools coated with PVD methods have a far longer service life than tools manufactured conventionally without protective coatings [5]. Coatings produced by the PVD process can be divided into simple (consisting of a single material, metal or phase) and complex, which consists of more than one material. Composite coatings include multi-component, multi-layer, multi-phase, composite and gradient coatings [6].

The application of protective coatings on tools for plastic working processes is primarily aimed at increasing wear resistance [7]. The wear process of plastic-forming tools carrying high mechanical and friction-wear loads is a complex phenomenon [8]. The process of tool wear consists of mechanical and thermal fatigue. Improper design of tools can be a source of stress accumulation, which initiates cracks under the influence of dynamic loads [9,10]. Another parameter-determining tool wear is the wrong selection of substrate material. Knowledge of the abrasion resistance of thin coatings can help in their correct selection for applications where abrasion plays a major role in their degradation [11]. The basic methods used to determine the durability of coatings are tribological tests using scratch tests [10] or tribotesters with various types: ball-on-disc [12], pin-on-disc [13], block-on-ring [14], etc.

Improving the anti-wear properties of AlTiN coatings has been the subject of many works over recent years. Wang et al. [15] found that the high coefficient of friction of AlTiN coatings was caused by the formation of a tribo-film on the wear track. Meng et al. [16] indicated that the synergistic effect of micro-textures and AlTiN coatings enhanced the anti-wear properties. The hybrid Al_x_Ti_1−x_N (x = ~0.65) coatings fabricated using cathodic arc evaporation and magnetron sputtering exhibited enhanced wear resistance and mechanical properties [17]. He et al. [18] investigated the tribological properties of the AlTiN coatings with different Al/Ti atomic ratios (73/27, 70/30, 67/33, 60/40, 50/50) and it was found that all coated inserts possessed an improved wear behaviour under wet cooling conditions. The indentation and wear tests on CrN-coated M50 disks indicated the relationships between applied loads and material removal patterns [19]. Biava et al. [20] experimentally analysed the high temperature corrosion behaviour of CrN, AlCrN and TiAlN coatings deposited by the arc evaporation PVD process onto Waspaloy Ni-based superalloy. The TiAlN and AlCrN and coatings showed a higher elastic modulus and hardness than the CrN coating. The wear investigations conducted by Navinšek and Panjan [21] indicated that a CrN coating (approx. 5 μm thick) with a Cr intermediate layer 0.2 μm thick between the substrate and the coating improved the quality of the surface finish of the products and increased tool life. Polok-Rubiniec et al. [22] found a correlation between hardness and adhesion of the CrN PVD coatings to the plasma-nitrided X37CrMoV5-1 and heat treated steel substrates. The analysis of the structure of CVD and PVD TiAlSiN coatings deposited on cemented carbide tools indicated that both coatings consisted of nanocrystals embedded in amorphous SiN_x_ [23]. Schulz et al. [24] deposited wear-resistive TiAlCrSiN coatings on a WC/Co metal substrate. The TiAlCrSiN coating showed a significant reduction in coefficient of friction due to the addition of Cr and Si to the coating which reduces adhesion. The wear behaviour of PVD coatings (TiN, TiAlN, TiC, TiAlN and Si_3_N_4_/AlTiN) in three ball-on-disc tests was analysed by Merklein et al. [25]. The TiC and TiAlCN coatings revealed a higher wear resistance than the coatings of AlCrN and TiN. The tribological performance of the AlTiN-TiSiN deposited by the PVD process was investigated by Claver et al. [26]. The experimental tests using the pin-on-disc test revealed that the combination of the nitriding process with the bonding layer deposited by high-power impulse magnetron sputtering improved the adhesion properties of AlTiN-TiSiN coatings. Das et al. [27] developed a nanocomposite AlTiSiN coating deposited using scalable pulsed-power plasma, which improved the surface finish of AISI D6 steel samples during turning. Liu et al. [28] investigated the microstructure and oxidation resistance of three nanocoatings (TiAlSiN/AlCrN multilayer, AlCrN monolayer and TiAlSiN monolayer). It was found that the AlCrN coating has the highest adhesion strength, while the TiAlSiN coating has the lowest adhesion strength among the coatings tested. Previous studies on AlCrN and TiAlSiN coatings were focused on thermal stability [29], high-temperature friction [30], microstructure [31], mechanical properties [32,33] and oxidation resistance [34,35]. Sousa et al. in a review paper [36] indicated the wear mechanisms of TiAlN-based coatings. Both AlCrN and AlTiN coatings are characterised by high oxidation resistance due to the formation of aluminium oxide surface layers [37].

This article attempts to characterise the coatings used on tools that form the pull-off caps made of EN AW-5052-H28 [38] aluminium alloy sheets. The technological process to produce food closures usually consists of precise cutting and stamping operations. The parameters of the input material, such as the coefficient of friction, are very important to ensure the proper course of the production process. In the food industry, particular emphasis is placed on product quality [39]. In this paper, the effect of the surface treatment of the EN AW-5052-H28 aluminium alloy sheets, consisting of varnishing the sheet metal surface using various varnishes and soaking the sheet metal, on the tribological properties, drawability, mechanical properties and wear resistance of test sheets is investigated. So far, there are no such studies available in the literature regarding EN AW-5052-H28 aluminium alloy sheets used in the food industry. The results of the tests showed differences in the mechanical and microstructural properties of the sheet depending on the method of varnishing. Based on the experimental results, significant differences in the adhesion of anti-wear coatings were found, despite the analogous method of preparing the steel substrate. The tests also showed fundamental differences in the microstructure of the coatings, and for some of them defects in the form of cracks parallel to the substrate and material losses were identified. The results of ball-on-disc tests concluded that the most favourable friction conditions among tested coatings are provided by the CrN coating. The low CoF of the CrN coating is associated with the smallest coefficient of volumetric wear of the sample and ball-shaped countersample.

The obtained results will allow for future research in industrial conditions aimed at determining the impact of the use of selected anti-wear coatings on the geometric quality of products and, consequently, on their application.

## 2. Materials and Methods

### 2.1. Test Material

**Figure 1:**
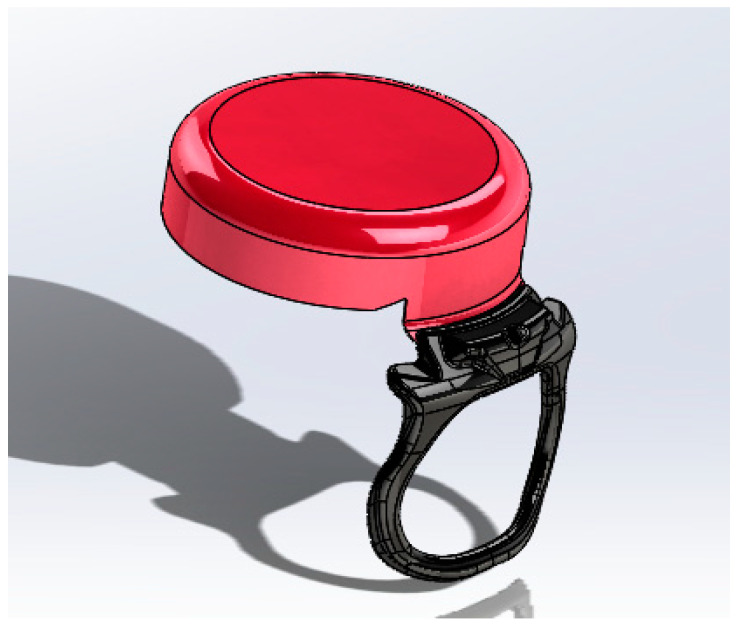
Exemplary geometry of lacquered pull-off cap.

The test material consisted of uncoated EN AW-5052-H28 aluminium alloy sheets with a thickness of 0.21 mm (Table 1), which were subjected to appropriate surface and/or heat treatment (Table 2). Selecting the appropriate sheet preparation technology is crucial in the manufacturing of pull-off caps (Figure 1) from the test material. The surface treatment consisted of varnishing the sheet metal surface using various varnishes and soaking the sheet metal at a temperature between 185 °C and 200 °C for 13 min. The inner surface of the pull-off cap was varnished. However, the outer surface was not varnished. To simplify the identification of lacquered samples, the sides of the sample are marked with colours: ‘white’ for non-lacquered surfaces and ‘yellow’ for lacquered surfaces. As a reference, the as-received sheet metal (sample no. 1 in Table 2) was used.

### 2.2. Surface Roughness Measurement

Surface roughness of the samples was measured on a laboratory stand equipped with a T1000 (Hommel-Etamic Jenoptik, Jena, Germany) roughness-measuring instrument. Surface roughness results are presented by the two most commonly used parameters, i.e., Ra—arithmetic average of the absolute values of the profile heights over the evaluation length and Rz—the average value of the absolute values of the heights of the five highest-profile peaks and the depths of the five deepest valleys within the evaluation length. The surface roughness measurement was carried out over a length of 4.8 mm. The test was performed for three orientations of the sample in relation to the sheet rolling direction: 0°, 45° and 90°. Five measurements were made for each direction. The tests were carried out for both sides of the analysed specimens.

### 2.3. Abrasion Resistance Roller-Block Test

**Figure 2:**
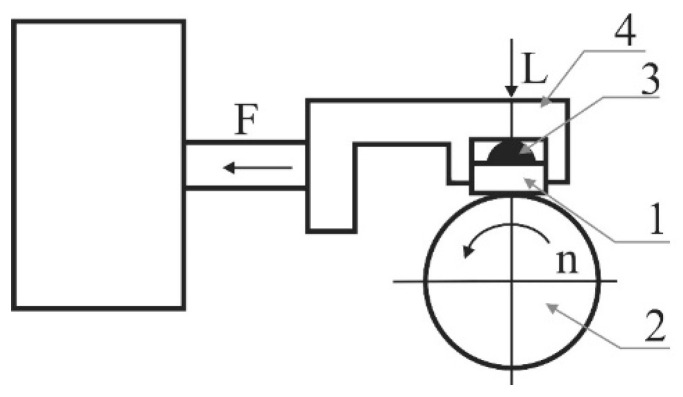
Principle of operation of T-05 roller-block tester: 1—block, 2—roller, 3—insert, 4—sample holder, F-friction force, L-load, n- rotation speed.

The abrasion resistance test of various grades of tool steels against the EN AW-5052-H28 aluminium alloy countersample was carried out on the T-05 roller-block tester. The abrasion resistance of samples presented in Table 2 was also tested. The measurement was carried out at an ambient temperature with translational motion in dry friction conditions. The principle of operation of the tester is shown schematically in Figure 2. The self-adjusting attachment of block (1), which is the sample holder (4) and the hemispherical insert (3), ensures that the block adheres to the roller (2) and evenly distributes the pressure on the contact surface. The tester used for the tests enables tests to be carried out in accordance with the ASTM D 2714, D 3704, D 2981 and G 77 standards. Countersamples from cold-work tool steels Caldie and Sverker 21 manufactured by Uddeholms AB (Hagfors, Sweden) were used in the tests.

The test samples were in the form of sheet metal strips with dimensions 20 × 4 × 0.25 mm. The average value of the average roughness Ra of the test samples, measured using a confocal microscope in accordance with ISO 21920-1:2021 [40], was 0.665 μm. Ring countersamples with a diameter of 49.5 mm made of Caldie and Sverker 21 steels applied with various coatings (Table 3) were used in the test. All measurements were performed at a constant ring rotational speed of 136 rpm. During the tests of the aluminium alloy samples (Table 2), a load of FN = 10 N was used and the friction path was 100 m. While testing the countersamples of Caldie and Sverker 21 steel, the load was 20 N and the friction path was 50 m.

During the abrasive test, the friction force FT was continuously recorded, which was used to determine the CoF µ according to Equation (1).

(1) $$\mu =\frac{F_{T}}{F_{N}}$$

The measure of abrasion resistance is the mass loss of the tested material in relation to the friction path and the applied load. The mass loss expressed in grams was determined in accordance with Equation (2), while the mass percentage of loss was determined in accordance with Equation (3).

(2) $$∆m=m_{p}-m_{k}$$

(3) $$∆m=\frac{m_{p}-m_{k}}{m_{p}}\cdot 100\%$$

where mp is the initial mass of the sample and mk is the final mass of the sample.

Observations of the surface morphology after friction test, measurements of surface roughness and identification of friction mechanisms were carried out using the LEXT OLS 4100 confocal microscope (Olympus Europe SE & Co. KG, Hamburg, Germany). The device is fully automatic in a simple, bottom-table arrangement for observation in reflected light. This microscope uses UV laser light with a wavelength of 405 nm.

### 2.4. Determination of the Coefficient of Friction (Ball-on-Disc Tribometer)

**Figure 3:**
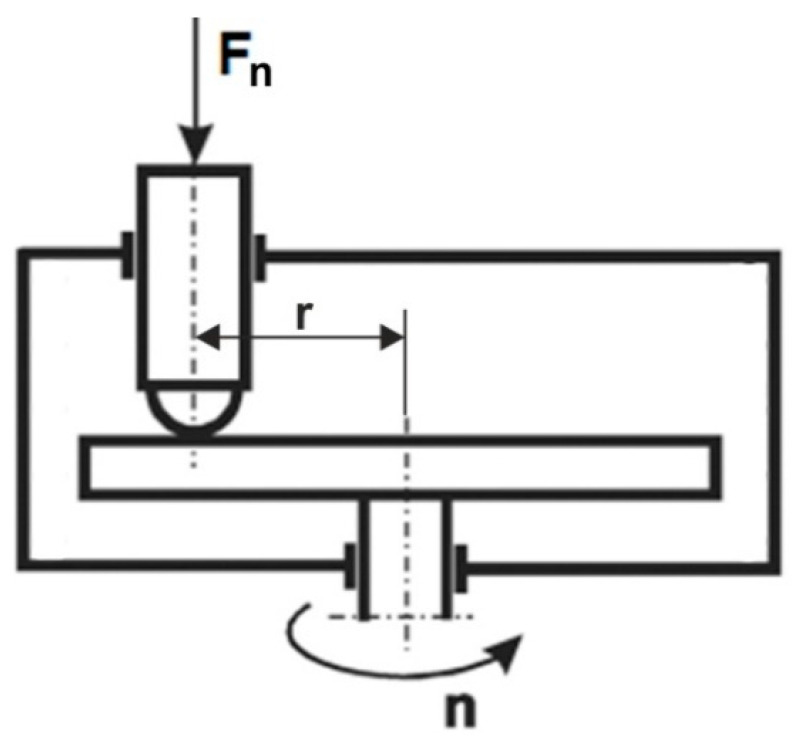
Schematic diagram of the ball-on-disc tribotester, Fn-pressure force, r-distance from the axis, n- rotation speed.

The CoF and the wear index were determined in accordance with the ISO 20808 standard. For this purpose, a T-21 ball-on-disc tribotester ((Łukasiewicz Research Network—Institute for Sustainable Technologies, Radom, Poland) designed to study the tribological properties of cooperating materials during sliding friction was used. The scheme of the T-21 ball-and-disc tribotester is shown in Figure 3. It consists of a rotating disc (sample) and a statically fixed countersample in the form of a pin with a spherical tip made of Al_3_O_4_. The pin is loaded with the force F.

During the test, the values of the friction force, rotational speed of the disc and depth of the wear track were recorded using a computer program. The normal load Fn is applied with weights placed on the countersample mounting lever. After the tests were completed, the samples were cooled to the ambient temperature and cleaned, and then the wear tracks were examined with a profilometer to determine the coefficient of volumetric wear Wv according to the Equation (4):

(4) $$W_{v}=\frac{V}{F_{n}\cdot s}\left[\frac{{mm}^{3}}{N\cdot m}\right]$$

where V is volume of the used material [mm^3^] and s is friction path [m].

The test was carried out with the following parameters: normal force Fn = 5 N, number of sample revolutions—15,000, radius of friction path r = 4, 5 and 6 mm. The volume of the worn material was determined based on the measurement of the cross-sectional area of the wear track. Measurements of the track profile after wear tests were carried out using an optical interferometric profilometer ProFilm3D (Filmetrics, San Diego, CA, USA). 

### 2.5. Mechanical Properties

The basic mechanical properties of the sheet metals were determined in a static tensile test using the Zwick/Roell Z020 uniaxial tensile testing machine (Zwick/Roell, Ulm, Germany). The tests were carried out in accordance with the EN ISO 6892-1:2022 standard [41]. The tests were carried out for samples cut in the following directions: perpendicular, parallel and at an angle of 45° to the direction of sheet rolling. Three samples of each type were tested and average values of the mechanical parameters were determined.

### 2.6. Analysis of Microstruture

The analysis of the microstructure of the samples was carried out using a metallographic microscope Axio Vert.A1 Mat equipped with an Axiocam 305 (ZEISS, Jena, Germany) camera, a metallographic microscope GX51 (Olympus Europe SE & Co. KG, Hamburg, Germany) and a scanning electron microscope SU 70 (Hitachi Ltd., Tokyo, Japan). The analyses of the chemical composition in the form of maps of the distribution of elements and of micro-areas of individual materials were performed using the energy dispersion spectroscopy (EDS) method.

Samples for microstructural analysis were cut out and then positioned in epoxy resin by Struers (Copenhagen, Danmark). Samples for optical microscopy were ground with #220, #500, #800, #1200, #2000 and #4000 grit SiC papers. Then the samples were polished with MD MOL (Struers) woven wool metal-backed polishing cloths using diamond powders with diameters of 6 μm and 3 μm. Finally, the specimens were polished for 2 min using metal-backed coarse- and fine-ground MD cloths in the presence of a suspension of silicon dioxide, organic solvents and water OP-S (Struers).

### 2.7. Hardness Testing

Nanohardness tests were carried out using the Step 500 device (Anton Paar Gmbh, Ostfildern, Germany), which is equipped with the nanoindenter NHT3 module (nanohardness tester) meeting the requirements of the ASTM-E2546-15 standard [42]. Hardness was determined by the Berkovich method at a load of 20 and 50 mN. The standard Berkovich indenter geometry with a centerline-to-face angle of 65.3° was used.

## 3. Results

### 3.1. Surface Roughness of Samples

**Figure 4:**
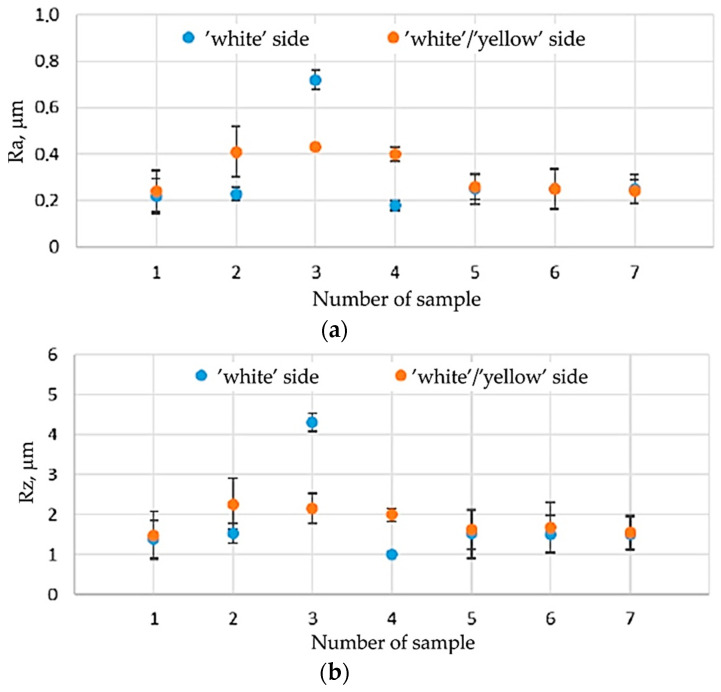
Average values of roughness parameters for the tested samples: (**a**) Ra, (**b**) Rz.

**Figure 5:**
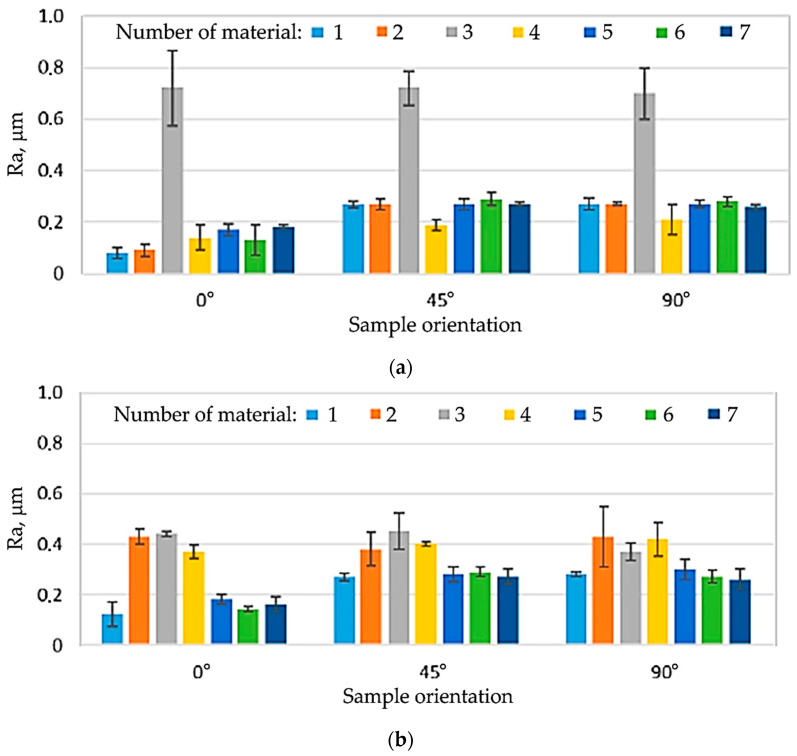
Values of the roughness parameter Ra depending on the orientation angle in relation to the sheet RD on (**a**) ‘white’ and (**b**) ‘white’/‘yellow’ sides.

**Figure 6:**
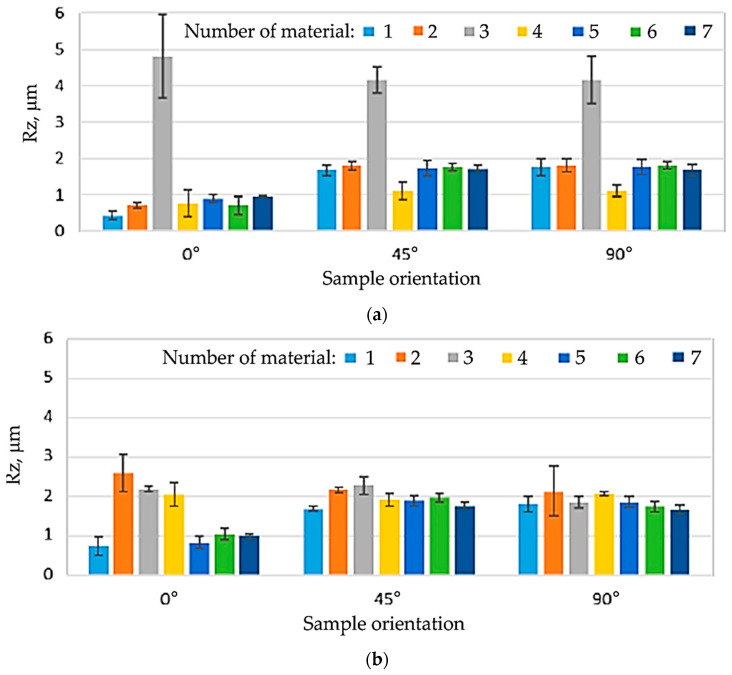
Values of the roughness parameter Rz depending on the orientation angle in relation to the sheet RD on (**a**) ‘white’ and ‘white’/‘yellow’ sides.

The tests showed significant differences in the surface roughness of the sample materials. It was found that the Ra and Rz roughness parameters of the analysed samples depend on both the material and the sample orientation in relation to the sheet rolling direction (Figure 4, Figure 5 and Figure 6). In the case of samples no. 2–4, the side (‘white’ or ‘yellow’) of the sheet that was tested was also important. In general, it was found that, in the case of non-lacquered materials (samples no. 1, 5–7), the average values of the roughness parameters on both sides of the sheet are similar (Figure 5). For samples no. 2 and 4, Ra and Rz were higher for the ‘yellow’ side, while the ‘white’ side of sample 3 was characterised by much higher roughness.

**Figure 7:**
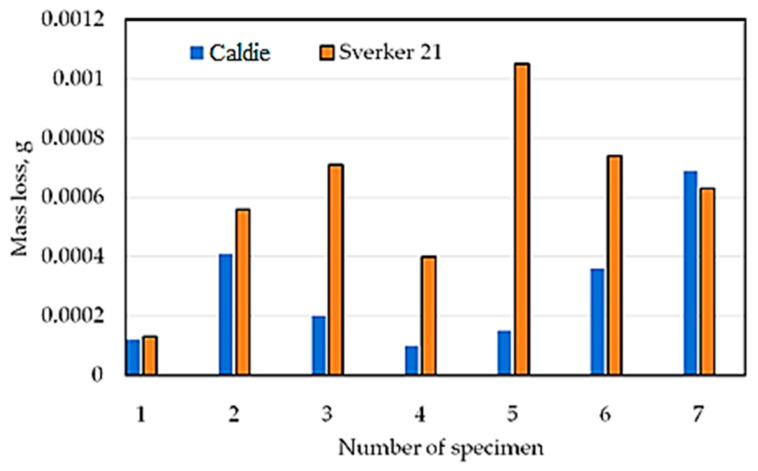
Mass loss of the countersample material.

For all the analysed sheets, a significant influence of the measurement direction on the value of surface roughness parameters was found, especially for the side without varnish. For samples no. 1 and 5–7, this relationship was observed for both non-lacquered sides of the samples. The lowest values of roughness parameters were recorded for the orientation 0° in relation to the sheet rolling direction (RD). For the remaining orientations (45°, 90°), the values of Ra and Rz were similar (Figure 6 and Figure 7). The values of surface roughness parameters of the lacquered samples no. 2–4 was different from the other samples and differed depending on the surface of the samples tested. In the case of sample 3, it can be concluded that there are no significant differences in the values of the Ra and Rz parameters for the tested directions in relation to the RD. Only slightly lower values of roughness parameters were observed for the 90° orientation and the ‘yellow’ side of the sample. In the case of samples no. 2 and 4, lower values of roughness parameters were found for the RD and the ‘white’ side of the sheet. On the other hand, for the ‘yellow’ side, the values of Ra and Rz were close to each other.

The tested sheets, especially without the varnish layer (samples no. 1, 5–7), are characterised by different values of roughness parameters depending on the orientation relative to the sheet RD. The surface roughness of the lacquered sheets (samples no. 2–4) was greater than that of the non-lacquered surfaces, although the distribution of Ra and Rz values on their surface was more even. The transparent varnish (Salchi VE2028) causes a significant increase in the surface roughness of the tested material, regardless of the orientation angle in relation to the sheet RD. For this situation, the Ra and Rz parameter values are also the highest. To sum up, the application and curing of the varnish have a significant impact on the surface roughness of samples.

### 3.2. Wear Resistance and Coefficient of Friction (Roller-Block Test)

**Figure 8:**
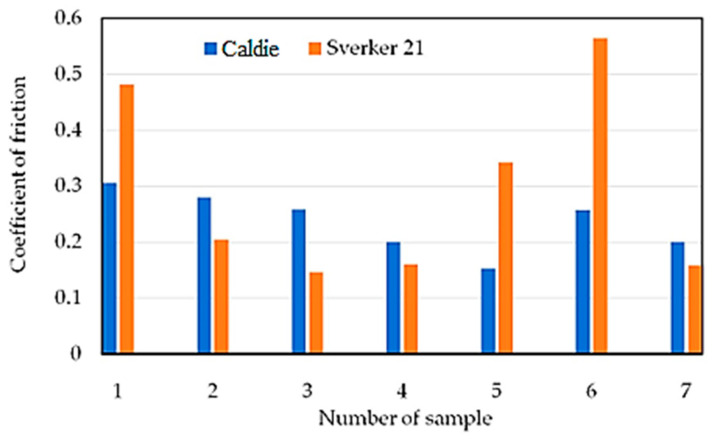
Average CoF of tested samples.

**Figure 9:**
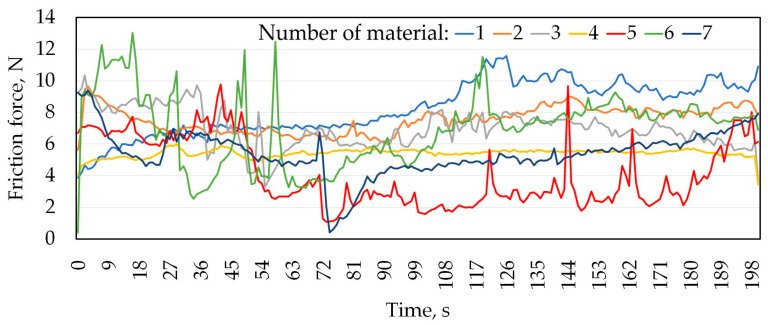
Changes in friction force as a function of test time for the Caldie steel countersample.

**Figure 10:**
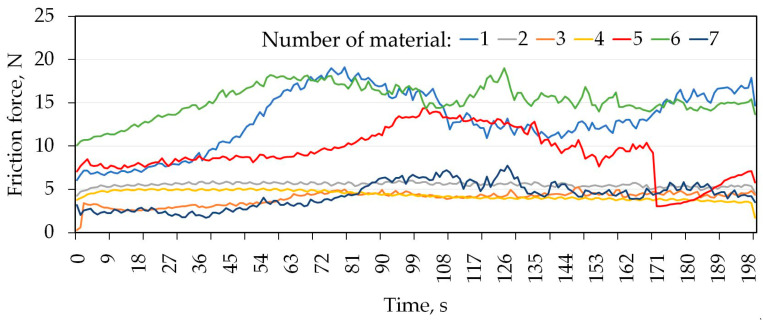
Changes in friction force as a function of test time for the Sverker 21 steel countersample.

Table 4 shows the mass loss of the materials under a load of 20 N. In addition, the results of mass loss and average CoF are shown graphically in Figure 7 and Figure 8, respectively. Analysing the presented results, for most of the tested sheets (samples 1–6), smaller mass losses were clearly recorded for the countersample of Caldie steel. Samples 1 and 5 without a varnish coating and sample 4 with a varnish coating are characterised by the lowest wear. The greatest difference in the CoF, approximately 50%, when testing with analysed countersamples, was observed for samples no. 5 and 6 (Figure 9). In turn, the smallest difference in the value of the CoF was observed for samples no. 4 and 7. Figure 9 and Figure 10 show the variation of the friction force during friction testing. In the case of Caldie steel countersample the friction force is at the most stable level and reaches the lowest values for sample 4. On the other hand, the highest CoF occurs in the case of contact of samples no. 1 and 6 with the countersample made of Sverker 21 steel. Test results with the Sverker 21 steel countersample (Figure 10) appear to show a more stable friction process compared with the Caldie steel countersample (Figure 9). For most of the samples (no. 2, 3, 4 and 7), the value of the friction force varies slightly throughout the test period and is much lower than for the as-received sheet metal (sample no. 1). No clear relationship was observed between the surface roughness of the samples and the course of the friction process.

**Figure 11:**
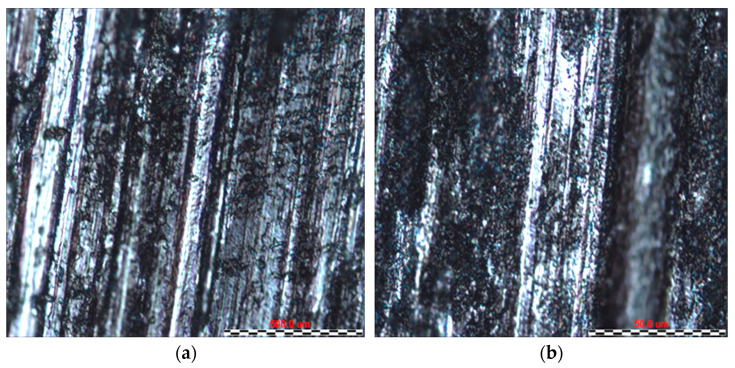
The surface of sample no. 1 after the friction process with countersample made of (**a**) Caldie and (**b**) Sverker 21 steel.

**Figure 12:**
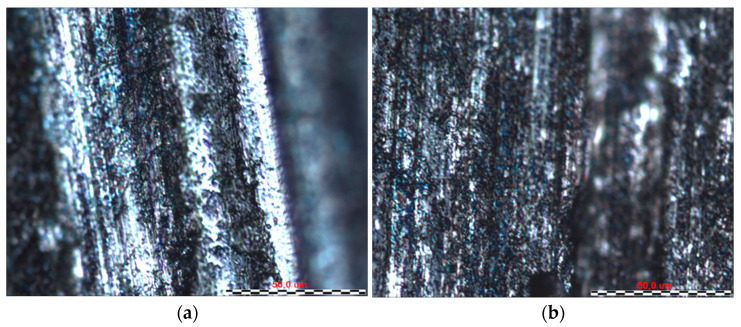
The surface of sample no. 2 after the friction process with countersample made of (**a**) Caldie and (**b**) Sverker 21 steel.

**Figure 13:**
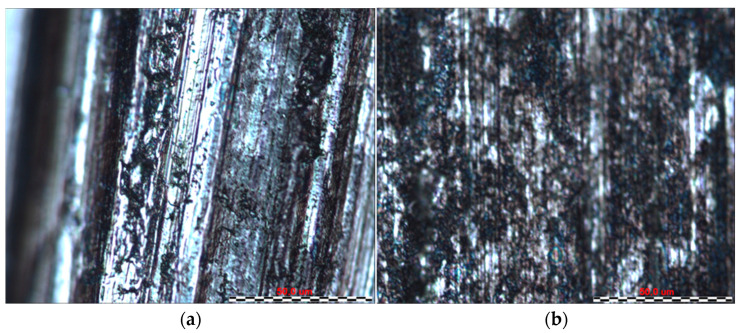
The surface of sample no. 3 after the friction process with countersample made of (**a**) Caldie and (**b**) Sverker 21 steel.

**Figure 14:**
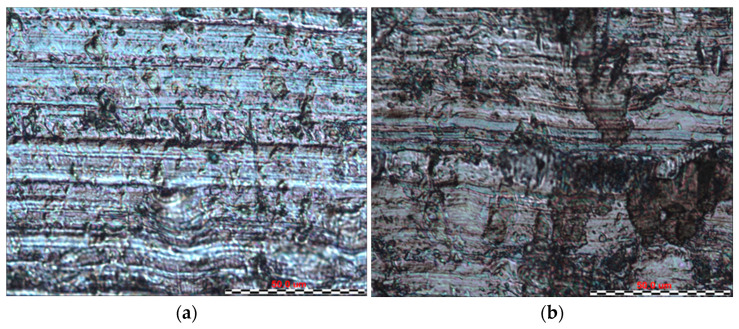
The surface of sample no. 4 after the friction process with countersample made of (**a**) Caldie and (**b**) Sverker 21 steel.

The course of changes in frictional force is more unstable for the Caldie steel counterexample; however, the lapping time is shorter for this counterexample, with a maximum of 72 s, where for the Sverker 21 steel it is up to 100 s. The Caldie steel counterexample caused deep scratches on the surface of the sheet, especially on painted samples. It seems to show some affinity with the varnish and cause it to detach from the surface, as the friction force stabilises after rubbing through the varnish. In some of the curves showing changes in frictional force as a function of test time, we observe abrupt changes in force over very short time periods, especially for tests with a countersample made of Caldie steel (Figure 9). This is due to the presence of aluminium in the friction node, which causes adhesion to occur. This mechanism is related to the formation and breaking of adhesive joints formed at the friction node. A stable course after degradation of the varnish layer is observed for samples no. 3 and 4 (Figure 9 and Figure 10). The behaviour of the samples in contact with Sverker 21 steel is completely different; only samples 1 and 5 show significant, abrupt changes in force during the entire tribological test. The surfaces of selected samples after the friction process, depending on the type of counterexample material, are shown in Figure 11, Figure 12, Figure 13 and Figure 14.

By analysing the friction surfaces shown in Figure 11, Figure 12, Figure 13 and Figure 14, it can be concluded that the typical wear mechanism in these materials is abrasive wear, with scratching and ploughing identified. Locally, adhesive wear and the accumulation of wear products in the form of sticker patches are also observed (Figure 11b). For sample 1, the Sverker 21 counterexample results in the accumulation of more wear products on the surface, which consist of oxides chipped from the surface. The main differences for samples 2 and 3 can be identified in terms of the depth of the scratches and grooves on the surfaces after friction (Figure 12 and Figure 13); deeper grooves were observed after using the counterexample made of Caldie steel. After using a counterexample made of Sverker 21 steel, the surfaces of the samples are ‘smoother’ and wear products adhering to the surface are also observed. Sample no. 4, for which the friction surfaces are summarised in Figure 14, differs from those presented in Figure 11, Figure 12 and Figure 13. In addition to the scratches, areas that are locally deformed are visible. This may be related to the disruption and overspray of the painted layer. This phenomenon is more pronounced for countersamples made of Caldie steel. Similar wear mechanisms after analysing the surfaces of the specimens were observed for unpainted specimens no. 5–7, where the dominant wear mechanism was abrasion of the sheet surface through scratching and grooving.

To sum up, depending on the method of surface preparation of the samples and the grade of countersample material, fundamentally different results of mass loss and CoF were obtained. The course of changes in the friction force is less stable for the Caldie steel countersample, while a stable course after rubbing through the varnish layer was observed for samples no. 3 and 4. In the case of samples no. 1–6, smaller mass losses were recorded for the Caldie steel countersample. Non-lacquered samples no. 1 and 5 and sample no. 4 exhibited the lowest wear. The CoF is stable and reaches the lowest values for sample no. 4 for both countersample materials. The highest CoF occurs when samples no. 1 and 6 meet the Sverker 21 steel countersample.

### 3.3. Mechanical Properties

**Figure 15:**
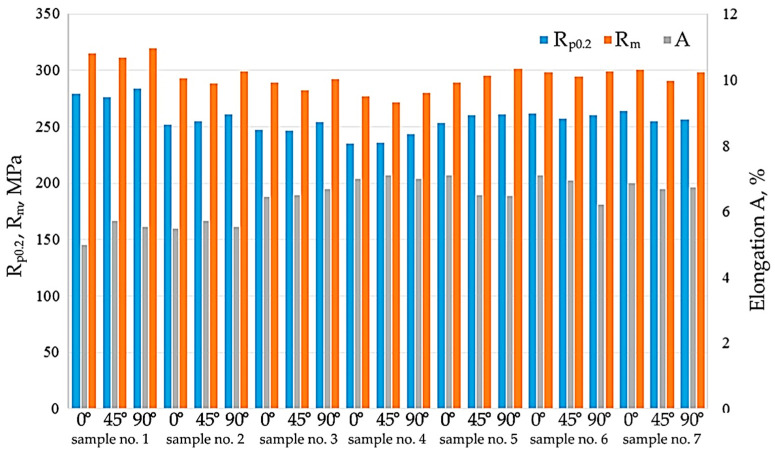
Average mechanical parameters obtained for all samples.

Figure 15 presents the results of the mechanical properties of sheets cut perpendicular, parallel and at an angle of 45° to the RD. Depending on the treatment method, the yield stress and ultimate tensile strength varied between 235.3 and 264 MPa, and between 272 and 301.3 MPa, respectively. However, all samples showed a decrease in these parameters compared to the as-received sample (sample no. 1). As-received material is characterised by the following mechanical parameters: yield stress Rp_0.2_ = 279.3, ultimate tensile strength Rm = 315 MPa and elongation A = 5.4%. The highest mechanical properties are exhibited by sample no. 1, and the lowest by sample no. 4 which was soaked at 200 °C. So, soaking has been shown to reduce the strength of EN AW-5052-H28 aluminium alloy sheet. All sheets are characterised by high anisotropy, with the lowest tensile strength being shown by samples cut at an angle of 45° to the RD and the highest by sheets cut perpendicular to the RD.

### 3.4. Hardness of Coatings

Coatings with high hardness and low modulus of elasticity can carry a significant load that does not plasticise the coating [14]. Ensuring both high hardness and hardness to Young’s modulus ratio with adequate substrate stiffness is particularly important for thin coatings; however, in their tribological applications, the value of the CoF should also be considered.

**Figure 16:**
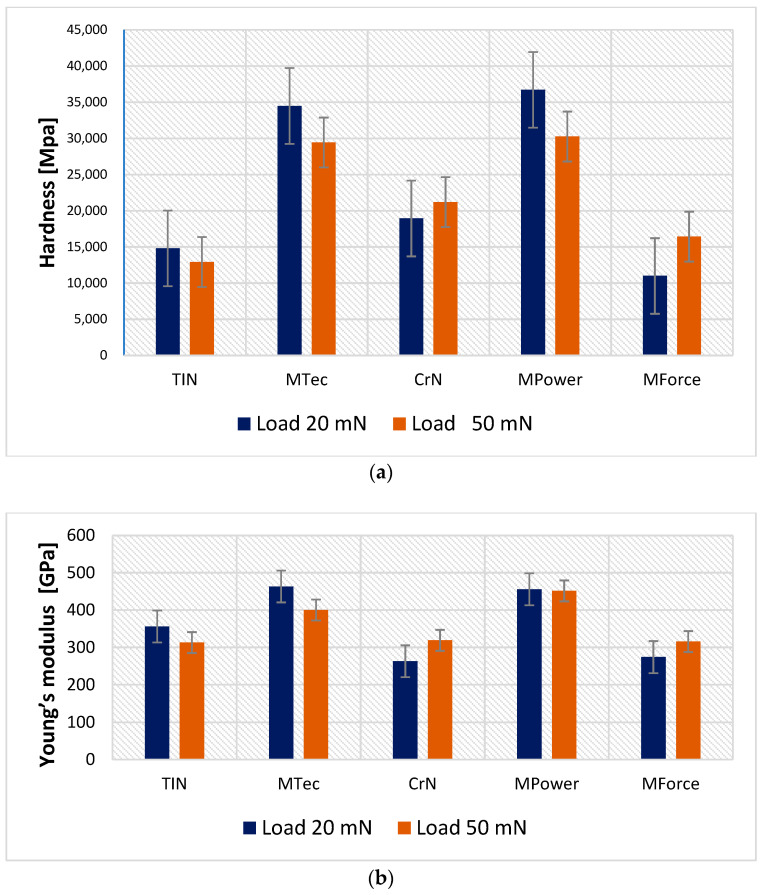
Hardness (**a**) and Young’s modulus (**b**) of the coatings at the maximum loading force of 20 mN and 50 mN.

For the assumed indenter loads of 20 and 50 mN, the penetration depth loads were significantly less than 1/10 of the coating thickness, which allows us to conclude that the obtained values are the properties of the tested coatings and the substrate had no effect on deformations. All coatings with the exception of MForce are characterised by greater hardness than the reference TiN coating. For the MPower shell, this value is more than twice as high. However, their Young’s modulus is slightly higher. This indicates that the H/E ratio will be higher for the proposed coatings, and thus the expected wear resistance will also be higher. This is confirmed by the results of tribological tests presented in the further part of the work in Figure 16. For the applied loads of 20 and 50 mN, no coating cracks were observed, which is also evidenced by the lack of pop-ins on the indentation curves.

### 3.5. Coefficient of Friction and Wear Coefficient (Ball-on-Disc Test)

**Figure 17:**
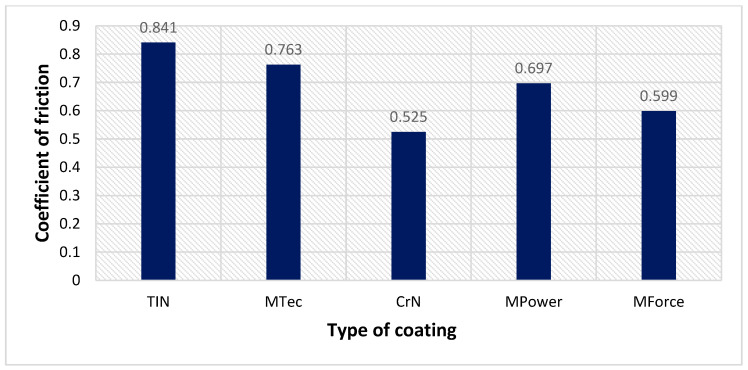
Average values of the CoF for analysed coatings.

**Figure 18:**
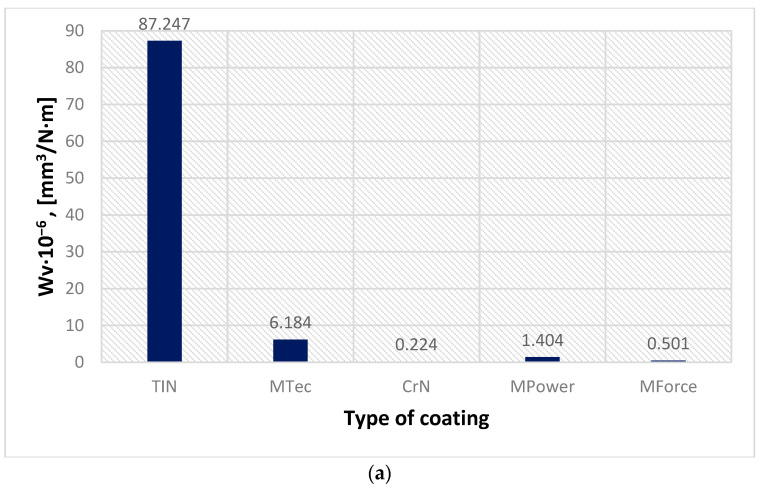

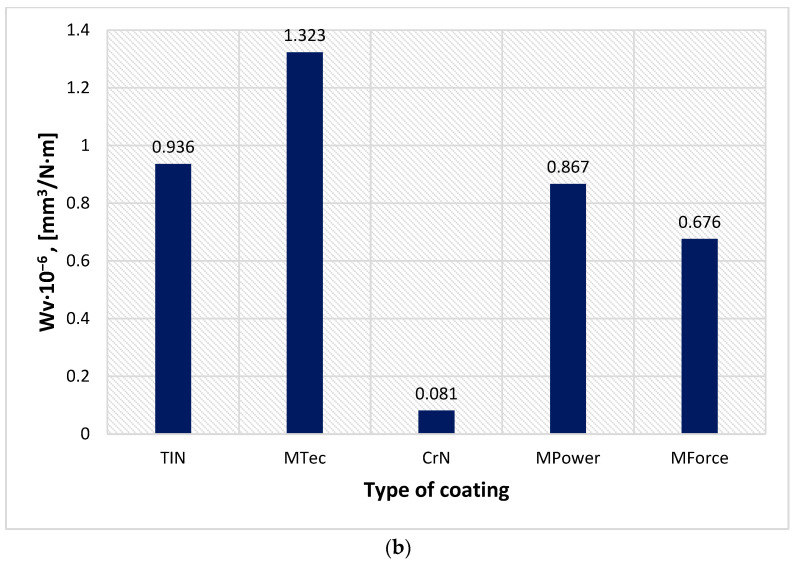
Coefficient of volumetric wear of the samples (**a**) and of the countersample (ball) (**b**).

Figure 17 presents a summary of the average values of the CoF of the analysed coatings. The highest value of the average CoF was observed for samples coated with CVD-TiN and MTec coatings. The most favourable friction conditions are provided by the CrN coating, for which the value of the CoF is about 36% lower than that of the CVD-TiN coating. The low CoF of the CrN coating is also associated with the smallest coefficient of volumetric wear of the sample (Figure 18a) and countersample (Figure 18b).

The sample coated with CVD-TiN showed the highest coefficient of wear index (87.2476·10–6 mm^3^/Nm). The coefficient of volumetric wear of the remaining coatings was less than 6.2·10–6 mm^3^/Nm. The value of volumetric wear for MPower, MTec, MForce and CrN coatings corresponds well with the value of volumetric wear of countersample (ball). The greater the value of volumetric wear of the sample (Figure 18a), the greater the value of volumetric wear of the countersample (Figure 18b). This relation does not apply to the CVD-TiN countersample. Despite the high value of the volumetric wear of this countersample, the ball wears at a similar level as during the wear test with MPower coatings. This proves the low wear resistance of the CVD-TiN coating. The greatest wear of the countersample concerned cooperation with the sample coated with MTec (1.3233·10–6 mm^3^/Nm).

**Figure 19:**
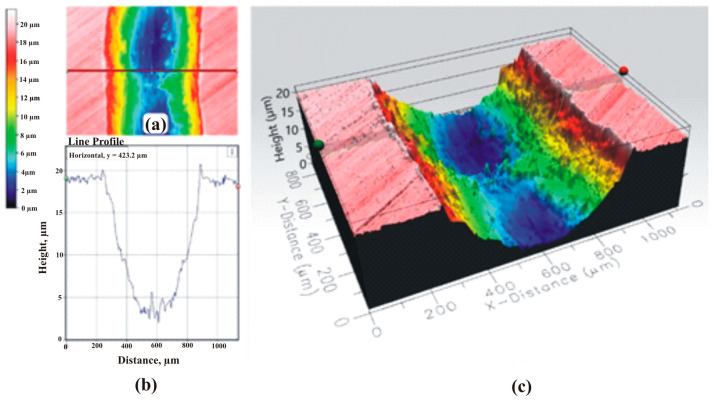
Topography of wear track (top view) (**a**), profile of friction track and (**b**) topography of friction track (isometric view) (**c**) for CVD-TiN sample.

**Figure 20:**
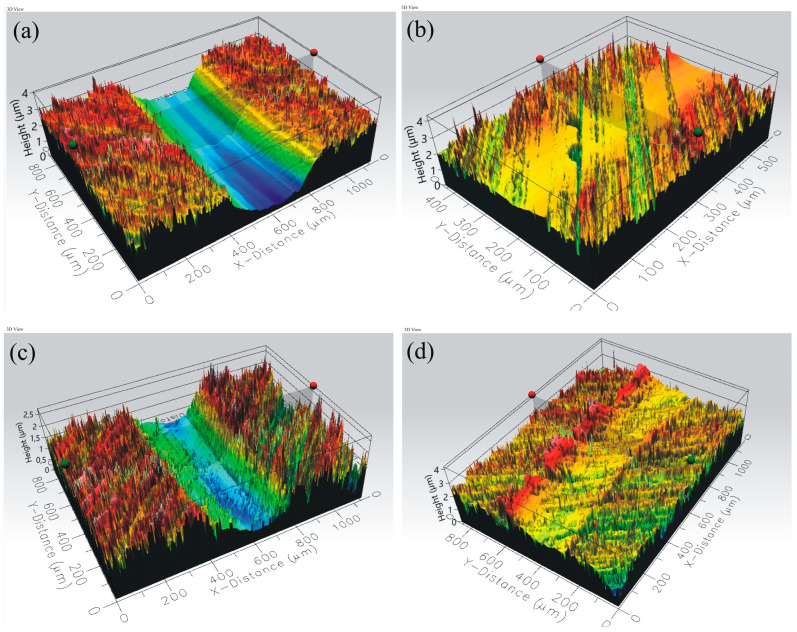
Three-dimensional topographies of friction track for samples coated with the following: (**a**) MTec, (**b**) CrN (**c**) Mpower, (**d**) Mforce.

Topographies of friction tracks of the coated samples are shown in Figure 19 and Figure 20.

### 3.6. Wear Resistance of Countersamples (Pin-on-Disc Tribometer)

Several countersamples with different properties in tribological contact with samples made of EN AW-5052-H28 aluminium alloy sheet were tested. The materials were analysed for their wear resistance, CoF and the morphology of worn surfaces in conditions of dry friction. The tests were carried out at ambient temperature (about 21 °C) and at about 30% humidity.

**Figure 21:**
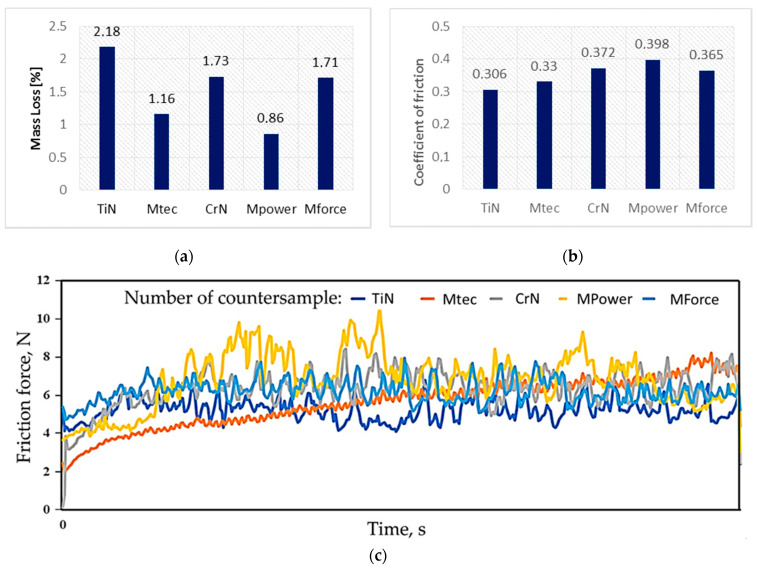
(**a**) Mass loss, (**b**) CoF and (**c**) variation of the friction force during the testing of coated countersamples against EN AW-5052-H28 aluminium alloy sheet.

Tribological tests were carried out using countersamples with as-received surfaces. The highest mass loss (2.18%) was observed for the countersample with the MTec coating. The countersamples coated with MForce and CrN coatings turned out to be the most wear resistant (Figure 21a). Despite the lowest mass loss, the CrN-coated countersample showed the highest CoF value, µ = 0.398 (Figure 21b). Among all the coatings tested, the difference in the CoF value is less than 10%. Despite fluctuations in the friction force, the countersamples coated with MTec, MPower Nano, CrN and MPower exhibited a stable average friction force throughout the test. Only the MForce-coated countersample showed a continuous increase in the friction force over the entire test period. This may indicate premature rupture of the coating and intensification of the wear processes. On the other hand, the value of the friction force during the test with this countersample was the most stable without large fluctuations.

The highest wear was recorded for the MTec-coated countersample at over 2% with a CoF of about 0.3. Practically no wear was observed on the surface of this countersample; there are trace scratches, while much greater abrasive wear from scratching and ploughing was found on the surface of the EN AW-5052-H28 aluminium alloy sheet. The course of changes in the friction force is quite unstable, and may be related to the accumulation of wear products on the surface of the sample. In the case of the MForce-coated countersample, its wear in contact with the EN AW-5052-H28 aluminium alloy sheet was insignificant and was within the measurement error limit, while almost the smallest mass loss of the countersample was recorded at over 1.16% with a slightly higher CoF (0.33) than in the case of the MTec-coated countersample.

A high coefficient of friction can increase wear on stamping tools, which increases production costs. Tools must be wear-resistant and durable to maintain the efficiency of the stamping process. In addition, the low coefficient of friction will improve the tool’s guidance due to a reduction in the heat released during work.

### 3.7. Microstructure

**Figure 22:**
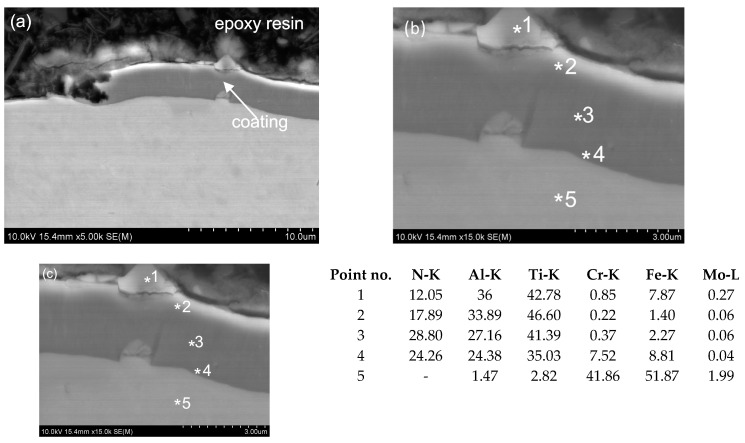
Microstructure of the MTec coating and substrate (SEM) (**a**), thickness of coating (**b**) and chemical composition (wt. %) of the MTec coating and the substrate (EDS-SEM) (**c**).

**Figure 23:**
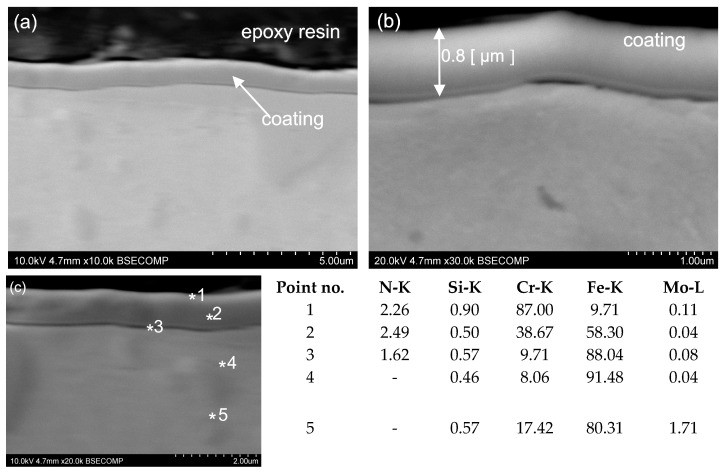
Microstructure of the CrN coating and substrate (SEM) (**a**), thickness of coating (**b**), chemical composition (wt. %) of the CrN coating and the substrate (EDS-SEM) (**c**).

**Figure 24:**
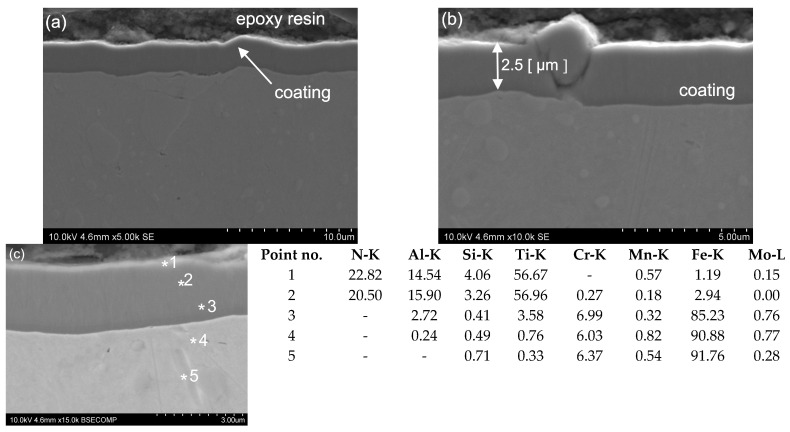
Microstructure of the MPower coating and substrate (SEM) (a), thickness of coating (**b**), chemical composition (wt. %) of the MPower coating and the substrate (EDS-SEM) (**c**).

**Figure 25:**
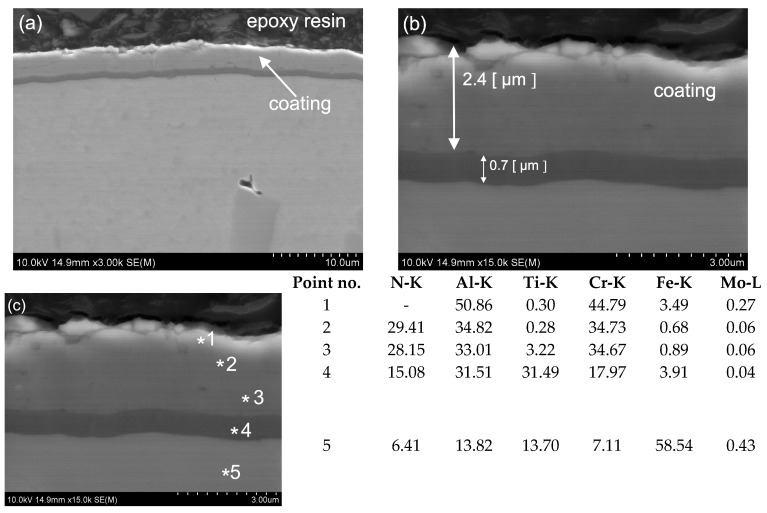
Microstructure of the MForce coating and substrate (SEM) (**a**), thickness of coating (**b**), chemical composition (wt. %) of the MForce coating and the substrate (EDS-SEM) (**c**).

The results of microstructural analyses of the tested coatings are summarised in Figure 22, Figure 23, Figure 24 and Figure 25. Using metallographic microscopy, thin protective coatings were identified on the surface of the substrate. In the case of the first tested sample, the MTec coating is unevenly deposited on the surface of the substrate, and it occurs only in small fragments over the entire area of the tested material (Figure 22). The SEM micrographs confirmed the uneven deposition of the coating on the surface of the steel substrate. Non-coated areas and some fragments of coating were identified (Figure 23). It should be noted that where the coating is present, it is homogeneous and adheres well to the surface of the substrate.

The thickness of the other two coatings, CrN and MPower, was, respectively, 0.8 and 2.5 µm (Figure 24 and Figure 25). The CrN (Figure 23) and MPower (Figure 24) coatings researched have a flat and smooth structure. Funnel-like artefacts were observed for the MPower coating locally owing to the presence of a droplet phase embedded in the coating, which is connected with the nature of the employed coating deposition CVD process (Figure 25). No microstructural defects were observed for either CrN or MPower coatings. In a few areas, for the CrN coating, delamination between the coating and the substrate was visible (Figure 23). Other areas where the coatings adhered well to the substrate surface are characterised by tight adherence to the substrate material. Small areas of delamination should not affect the quality and strength of the coating. This can be confirmed by the results of the study of the coefficient of friction and wear coefficient (Figure 18).

The MForce multi-layer coatings reveal dense structures with not-visible delamination and defects. The coating was uniformly distributed over the entire width of the test sample. For a more accurate microstructural analysis, SEM measurements were performed, confirming the presence of two superimposed layers. The thickness of the main layers of the MForce (AlTi)N/(Al,Ti)N coating were equal 0.7 and 2.4 µm (Figure 25). In the tested areas of the coating, the point analysis using an X-ray energy dispersive spectrometer EDS indicated the presence of such elements as Al, Ti and N (Figure 22c, Figure 23c, Figure 24c, Figure 25c). The * symbol indicates the place of point analysis of chemical composition. Analysis of the chemical composition showed that the interlayer is rich in Ti, Al and N. The overlying coating contains Cr, Al and N. In the tested areas, the outer layer shows areas where microstructural defects in the form of cracks and material losses were identified. The coating shows good adhesion to the substrate surface.

## 4. Conclusions

This article presents the results of tribological and microstructural studies of coatings applied to tools for forming pull-off caps. As a test material, EN AW-5052-H28 aluminium alloy sheets with different surface treatments were used. Measurements of surface roughness of sheet metals and basic mechanical properties of sheet metals and hardness of coatings, as well as wear tests and formability tests using the Erichsen method, were carried out. Based on the comprehensive tribological and microstructural investigations, the following conclusions can be drawn:

- It was found that the Ra and Rz roughness parameters of the analysed samples depend on both the material and the sample orientation in relation to the sheet RD. The lowest values of roughness parameters were recorded for the orientation 0° in relation to the sheet RD.
- For most of the tested sheets (samples 1–6), smaller mass losses were recorded for the countersample made of Caldie steel.
- Lacquered and/or soaked samples showed a decrease in yield stress and ultimate tensile strength compared to as-received sample. Samples are characterised by high anisotropy, with the lowest tensile strength being shown by samples cut at an angle of 45° to the RD and the highest by sheets cut perpendicular to the RD.
- Based on SEM micrographs and EDS mapping, it was found that the microstructure of the test sheets is characterised by the existence of precipitates that are rich in Fe, Si, Mn, Mg and Si.
- The results of ball-on-disc tests concluded that the most favourable friction conditions are provided by the CrN coating. The low CoF of the CrN coating is associated with the smallest coefficient of volumetric wear of the sample and ball-shaped countersample.
- The tests of the chemical composition using the EDS method confirmed the composition of all applied coatings.
- The microstructure of the MForce coating is two-layered, and the analysis of the chemical composition confirmed the presence of (AlTi)N/(AlCr)N layers.
- The (AlTi)N interlayer in the MForce coating adheres to the substrate over the entire tested area and no detachment from its surface was observed, which proves good bonding at the substrate/coating interface.
- Metallographic tests showed that, in the case of the (AlTi)N coating, there are defects in the form of discontinuities.
- In the tested areas of the MForce (AlTi)N/(AlCr)N coating, defects in the form of cracks parallel to the substrate and material losses were identified.
- The tested CrN and MPower (AlTiN/TiAlSiXN) coating is characterised by a homogeneous, compact microstructure.
- The occurrence of funnel-shaped artefacts resulting from the existence of a droplet phase embedded in the coating was found. A few areas with delamination between the coating and the countersample substrate were also observed.
- Future work will include research in industrial conditions on the impact of the use of anti-wear coatings selected on the basis of tests of the geometric quality of products.

## Figures and Tables

**Table 1:** Chemical composition [% wt.] of the EN AW-5052-H28 aluminium alloy.

Si	Fe	Cu	Mn	Mg	Cr	Zn	Ti
0.4	0.5	0.1	0.5–1.1	1.6–2.5	0.3	0.2	0.1

**Table 2:** Parameters of surface treatment of samples.

Number of Sample	Lacquering			Soaking Temperature and Duration			Colour of Surface	
	Adhesive Varnish Salchi VI 1106	Coating Varnish Salchi ANC 6001	Overprint Varnish Salchi VE2028	200 °C, 13 min	190 °C, 13 min	185 °C, 13 min	Inner Side	Outer Side
1	no	no	no	no	no	no	‘white’	‘white’
2	yes	no	no	yes	no	no	‘yellow’	‘white’
3	yes	yes	no	yes	yes	no	‘yellow’	‘white’
4	yes	yes	yes	yes	yes	yes	‘yellow’	‘white’
5	no	no	no	yes	no	no	‘white’	‘white’
6	no	no	no	yes	yes	no	‘white’	‘white’
7	no	no	no	yes	yes	yes	‘white’	‘white’

**Table 3:** Types of coatings used on Caldie and Sverker 21 steel samples.

Sample Designation	Type of Coating
PR1	MTec (AlTi)N
PR2	CrN
PR3	MPower (AlTiN/TiAlSiXN). X = Cr, B, Y
PR4	MForce ((AlTi)N/(AlCr)N)

**Table 4:** Mass loss of test countersamples.

Sample Number	Countersample Material							
	Caldie				Sverker 21			
	m_1_, g	m_2_, g	Δm, g	Δm, %	m_1_, g	m_2_, g	Δm, g	Δm, %
1	0.03599	0.03587	0.00012	0.33	0.03940	0.03927	0.00013	0.33
2	0.04083	0.04042	0.00041	1.00	0.03879	0.03823	0.00056	1.44
3	0.03865	0.03845	0.0002	0.52	0.04063	0.03992	0.00071	1.75
4	0.04148	0.04138	0.0001	0.24	0.03993	0.03953	0.0004	1.00
5	0.03746	0.03731	0.00015	0.40	0.03548	0.03443	0.00105	2.96
6	0.03593	0.03557	0.00036	1.00	0.03388	0.03314	0.00074	2.18
7	0.03800	0.03731	0.00069	1.82	0.03693	0.03630	0.00063	1.71

## Data Availability

The data presented in this study are available on request from the corresponding author.
